# Synthesis of Carbon Nanomaterial from Coke and Preparation of Copper Oxide-Based Composite

**DOI:** 10.3390/molecules31122129

**Published:** 2026-06-17

**Authors:** Zhanar Assirbayeva, Zhazira Mukatayeva, Nurgul Shadin, Yerbol Tileuberdi, Qiang Zeng, Aigul Nurakhmetova, Khanat Dyussebayev, Klara Sarsekova, Yrysgul Bakytkarim

**Affiliations:** 1 Department of Chemistry, Faculty of Natural Sciences and Geography, Abai Kazakh National Pedagogical University, 13, Dostyk Ave., Almaty 050010, Kazakhstan; asirbaeva.j88@gmail.com (Z.A.); zh.mukatayeva@abaiuniversity.edu.kz (Z.M.); er.tileuberdi@gmail.com (Y.T.); nurakhmetovaa@gmail.com (A.N.); khanat581219@mail.ru (K.D.); k.sarsekova@mail.ru (K.S.); 2 School of Chemistry and Chemical Engineering, South China University of Technology, Guangzhou 510641, China

**Keywords:** coke-derived carbon, petroleum coke, nanocomposite, copper oxide, electrochemical impedance spectroscopy, dichlorvos, electrochemical sensor

## Abstract

The development of low-cost and highly sensitive electrochemical sensing platforms for pesticide monitoring has attracted significant attention in recent years. In this study, coke-derived carbon (CDC) was successfully synthesized from petroleum coke through high-temperature carbonization under a nitrogen atmosphere. Subsequently, a CDC@CuO-NP nanocomposite was fabricated by depositing copper oxide nanoparticles onto the CDC matrix. The morphology, structure, and elemental composition of the synthesized materials were characterized using scanning electron microscopy (SEM), transmission electron microscopy (TEM), energy-dispersive X-ray spectroscopy (EDS), and elemental mapping analyses, confirming the successful formation of the composite and the uniform distribution of CuO nanostructures on the carbon surface. Electrochemical characterization demonstrated that the incorporation of CuO significantly enhanced the electrochemical performance of CDC by increasing the electroactive surface area and facilitating electron transfer. The CDC@CuO-NP-modified glassy carbon electrode was applied for the electrochemical detection of dichlorvos (DDVP) using electrochemical impedance spectroscopy (EIS). The sensor exhibited a concentration-dependent increase in charge-transfer resistance and showed a linear response in the concentration range of 247–3770 nM, with the regression equation y = 47.1458C + 111.8162 and a correlation coefficient of R^2^ = 0.9832. The developed sensor achieved a low limit of detection (LOD) of 2.3 nM, demonstrating high sensitivity toward DDVP. These results indicate that the CDC@CuO-NP nanocomposite is a promising, low-cost, and efficient electrode material for the sensitive determination of organophosphorus pesticides and has considerable potential for environmental monitoring and food safety applications.

## 1. Introduction

Petroleum coke (PC) is a carbon-rich by-product of the petrochemical industry and has recently gained attention as a low-cost, readily accessible precursor for the synthesis of carbon nanomaterials. Recent research has shown that petroleum coke can be successfully transformed into a variety of nanostructured carbon materials, such as graphene-like materials and reduced graphene oxide, with high electrical conductivity and high specific surface area. Due to these unique properties, petroleum coke-based carbon materials have been considered promising candidates for the development of electrodes and electrocatalyst supports [1,2]. The properties and structure of carbon materials are highly dependent on the synthesis route and type of carbon precursor used. There are a variety of technological routes used for coke production depending on the type of requirement and its intended use [3].

Depending on their graphitization behavior, carbon materials emanated from petroleum coke are generally classified as amorphous carbons, including soft carbon and hard carbon. Hard carbon, in particular, is a non-graphitizable material represented by a highly disordered structure and improved interlayer spacing [4]. Usual antecedents for hard carbon include polymers, epoxy and synthetic resins, biomass-derived materials and conducting polymers such as polyaniline. Although hard carbon cannot be graphitized even at temperatures as high as 3000 °C [5], it has proven to be a promising anode material for sodium-ion batteries (SIBs). In recent years, coal-based solid carbon has attracted widespread attention as an anode material for sodium-ion batteries [6]. Today, coal-based hard nanocarbon is considered a promising anode material for sodium-ion batteries due to its high energy density, relatively low operating potential, and stable supply. In addition, coal has clear advantages in cost, production scalability, and commercialization. Several promising materials based on solid carbon from coal have been proposed and tested [7], but there remains a need for continuous improvement of this material due to its low initial Coulombic efficiency [8].

Carbon nanomaterials have a range of remarkable characteristics in several industrial sectors [9], among them carbon allotropes: graphite, diamond, graphene, oxidized (GO) and reduced (rGO) graphene, carbon nanotubes (CNTs), fullerenes, carbon (CQDs) and graphene (GQDs) quantum dots, and others. In this group of carbonaceous nanomaterials, graphene has attracted increasing interest in research in the most diverse scientific areas because of its excellent physicochemical, thermal, mechanical, and electrical properties, which make graphene one of the most studied CNMs [10,11,12,13,14,15,16,17]. However, obtaining it in a cheaper, industrially scalable way remains a challenge. Thus, using other more abundant, lower-cost resources, such as petcoke, can be a great alternative.

Carbon materials have high conductivity, large surface area, and high chemical stability, making them popular materials for electrochemical detection. The main disadvantages of traditional electrodes are their low sensitivity and poor selectivity. One of the most common methods for improving the performance of an electrochemical sensor is to modify the electrode with a high-surface-area nanomaterial. Carbon electrodes are well-suited for electrochemical detection due to their large specific surface area, uniform and controllable pore-size distribution, long-range structural order comprising mesoporous channels, and excellent conductivity [18]. In recent publications, many scientists have turned their attention to carbon-based nanocomposite materials as electrodes for various applications due to their significant advantages [19].

Metal oxides can be categorized into three classes based on their chemical properties and structural characteristics. These three main categories are acidic, basic, and amphoteric oxides [20]. Metal oxides are compounds composed of metal-bound oxygen atoms that exhibit admirable physicochemical properties. The metal oxides can be prepared in various sizes, ranging from the microscale to the nanoscale [21]. A metal oxide is considered a conventional model due to its excellent properties, including abundant active sites, large specific surface area, various electronic states, high electronic conductivity, enhanced electrochemical behavior, and thermal properties [22]. Copper oxide (CuO) nanostructures are of particular interest due to their interesting properties and promising applications in batteries, electric capacitors, solar cells, gas sensors, biosensors, catalysis, photodetectors, energy materials, field emissions, hydrophobic surfaces, and the removal of arsenic and organic pollutants from wastewater [23,24].

Previous studies have extensively investigated CuO and carbon nanotube (CNT)-based nanocomposites for the enhancement of optical properties. In particular, CNTs/CuO nanocomposites synthesized through chemical deposition and ultrasonic-assisted methods have been reported to exhibit increased light absorption and reduced reflectance. These characteristics make such nanocomposites promising candidates for applications in solar energy harvesting and conversion systems. In addition, copper oxide nanowires (CuO NWs) and single-walled carbon nanotubes (SWCNTs) [25], as well as 3DGR- and CuO-NP-based [26] hybrid nanocomposites, are effective modifiers for the electrochemical detection of organophosphate pesticides. Dichlorvos (DDVP) is one of the organophosphate pesticides widely used in agriculture and for controlling household pests [27]. DDVP inhibits the enzyme acetylcholinesterase, leading to nervous system dysfunction and other adverse physiological effects. Therefore, accurate and rapid determination of DDVP [28] levels in the environment and food products is one of the most pressing issues.

Therefore, the main objective of this work was to synthesize coke-derived carbon (CDC) from petroleum coke and develop a CDC@CuO nanocomposite with enhanced electrochemical properties. The synthesized composite was comprehensively characterized in terms of its morphology, structure, and elemental composition. In addition, the electrochemical performance of the CDC@CuO-modified electrode was investigated toward the detection DDVP using electrochemical impedance spectroscopy. The proposed approach combines the high conductivity and large surface area of CDC with the catalytic activity of CuO, providing a simple and cost-effective platform for the sensitive monitoring of organophosphorus pesticides.

## 2. Results and Discussion

### 2.1. Surface Morphology

**Figure 1:**
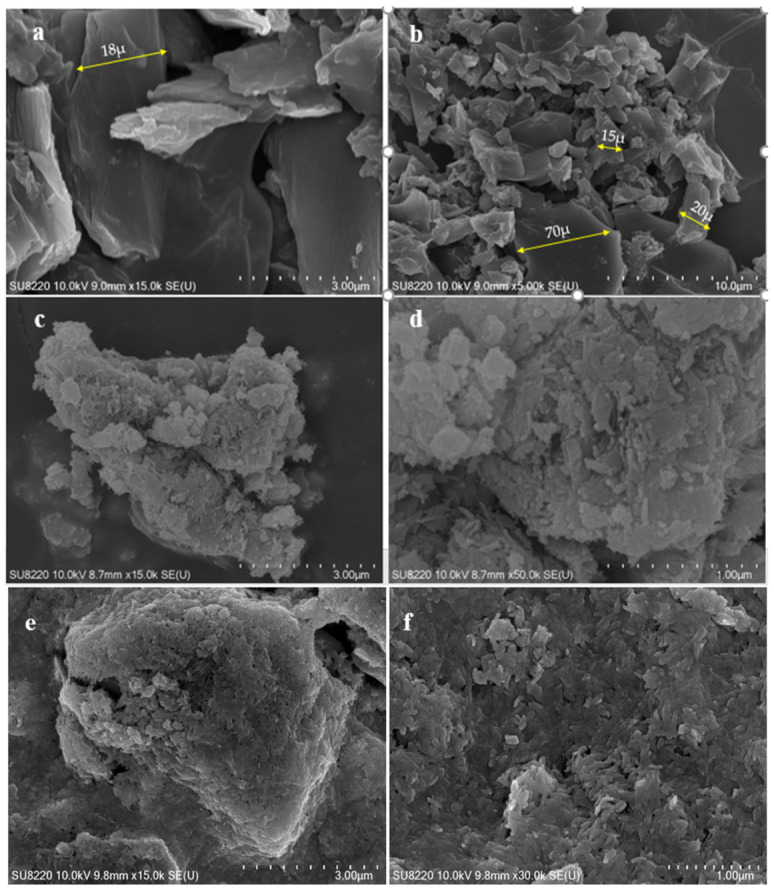
SEM images of (**a**,**b**) CDC, (**c**,**d**) CuO-NPs, and (**e**,**f**) CDC@CuO-NPs.

The morphology of CDC, CuO, and CDC@CuO-NPs was investigated using SEM, as shown in Figure 1. The CDC material (Figure 1a,b) is characterized by a layered, plate-like structure. In some areas, the sheets are neatly stacked on top of one another, while in other areas, the material has an amorphous, dispersed structure, with the sheets arranged randomly, possibly due to breakage or delamination of larger sheets. The morphology of the synthesized CuO is shown in Figure 1c,d. At low magnification (Figure 1c), CuO exhibits a highly agglomerated structure consisting of irregularly shaped clusters. These aggregates form from smaller primary particles, indicating a strong tendency for the particles to agglomerate. At higher magnification (Figure 1d), the surface of these aggregates appears rough and heterogeneous, consisting of densely packed granular and flaky nanostructures. However, the observed agglomeration may also affect the uniformity of particle distribution in composite systems. To determine the uniformity and distribution of CuO on the CDC surface, as shown in Figure 1e,f, low-magnification SEM images are used. The CuO matrices exhibit a more uniform distribution (Figure 1e), CuO is observed in the form of clusters of large nanofibers mixed with small NP (Figure 1f). CuO nanoparticles consist of two main components: continuous nanofibers with widths ranging from <20 to 700 nm and small rods.

**Figure 2:**
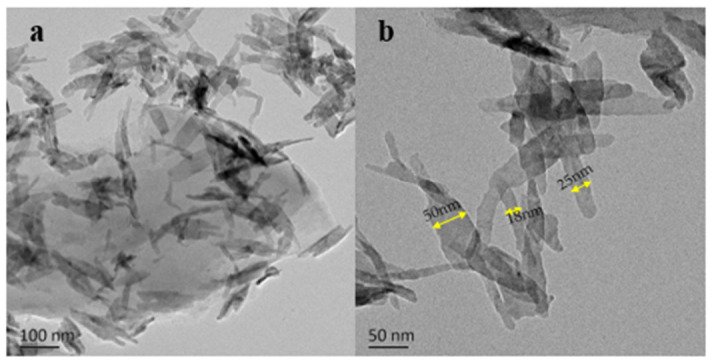
TEM images of (**a**) lower, and (**b**) higher magnification of CDC@CuO-NPs.

The microstructure of the CDC@CuO-NP nanocomposite was investigated by transmission electron microscopy (TEM), as shown in Figure 2. The images reveal a hybrid architecture consisting of a carbon-based matrix decorated with CuO nanostructures. At lower magnification (Figure 2a), the material exhibits a loosely aggregated network composed of thin, sheet-like and fibrillar features. These structures are randomly oriented and form an interconnected framework, indicating the disordered nature of the carbon support. At higher magnification (Figure 2b), CuO nanostructures are observed as semi-transparent nanosheets with irregular shapes and varying lateral dimensions. The nanosheets are ultrathin and partially overlapping, suggesting a high degree of dispersion across the carbon matrix. Their transparency indicates a small thickness, while regions of higher contrast correspond to stacked or folded layers. The intimate contact between CuO nanosheets and the CDC matrix suggests strong interfacial interaction, which is beneficial for efficient charge transfer. Such a morphology, combining conductive carbon with high-surface-area CuO nanosheets, is expected to significantly enhance electrochemical performance due to the synergistic effect of both components.

**Figure 3:**
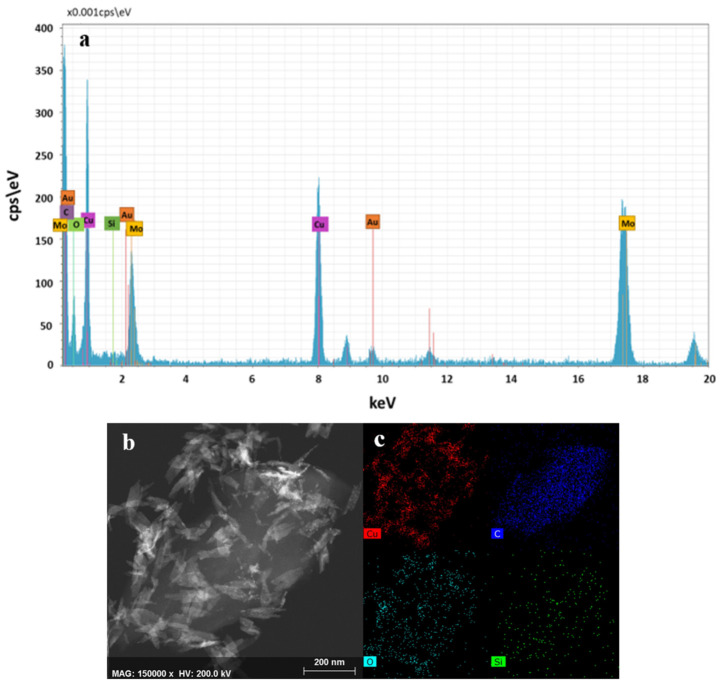
(**a**) Energy-dispersive spectroscopy, (**b**) TEM image, and (**c**) elemental mapping of the CDC@CuO nanocomposite.

Furthermore, the elemental composition of CDC@CuO-NPs was analyzed using energy-dispersive spectroscopy (EDS), as shown in Figure 3a. The spectrum indicates the presence of the main elements C, O, and Cu. A pronounced carbon (C) peak in the low-energy region indicates that carbon constitutes the main matrix of the CDC material, whereas the oxygen (O) signal corresponds to the oxidized phase. The characteristic Cu peaks observed in the range from approximately 0.9 keV (L line) to 8.0 keV (Kα) further confirm the successful incorporation of CuO into the carbon structure. In addition to the main elements, signals corresponding to molybdenum (Mo) were detected. These signals originate from the molybdenum support grid used during the TEM/EDS analysis and are unrelated to the sample’s internal composition. Furthermore, the presence of gold (Au) peaks is due to the sample surface being coated with a thin layer of gold before analysis. Such a coating is used to improve electrical conductivity and reduce charging effects during electron microscopy measurements. In addition, a weak silicon (Si) signal was also detected, the presence of which is explained by residual mineral impurities in the petroleum coke or the synthesis process. In addition, the mapping of elements also approves the distribution of the elements C, Cu, O, and Si present in CDC@CuO-NPs, as shown in Figure 3b,c. Finally, we conclude that the CuO-NPs are successfully deposited on the CDC structure.

### 2.2. Electrochemical Characterization of CDC and CDC@CuO Nanocomposites

**Figure 4:**
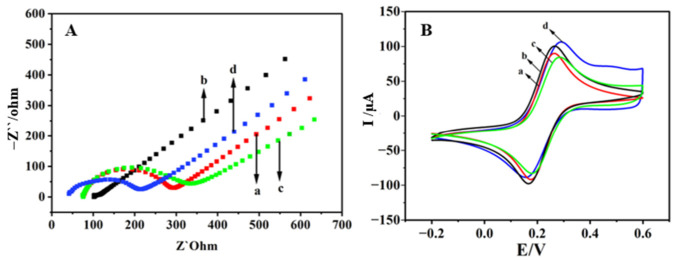
(**A**) The Nyquist plots of different modified electrodes in 5 mmol L^−1^ K_3_[Fe(CN)_6_]/K_4_[Fe(CN)_6_] and 0.1 mol L^−1^ KCl solution (pH 6.5), (**B**) CV curves of the bare GCE (curve a), CDC/GCE (curve b), CuO/GCE (curve c) and CDC@CuO-NPs/GCE (curve d) at a scan rate of 50 mV s^−1^.

The electrochemical characterization of the CDC/GCE and CDC@CuO/GCE sensor was carried out using EIS [29] and CV [30]. EIS is an effective analytical technique for studying the surface properties and interfacial behavior of modified electrodes, and CV is an important method for determining the active surface area of electrodes, especially suitable for nanomaterial systems, as this parameter plays a crucial role in characterizing their efficiency. Furthermore, cyclic voltammetry can also determine electrocatalytic activity and can be used to compare the efficiency of nanoparticle modified electrodes. The measurements were carried out in a solution containing 5 mmol L^−1^ K_3_[Fe(CN)_6_]/K_4_[Fe(CN)_6_] and 0.1 mol L^−1^ KCl. As shown in Figure 4A,B, the Nyquist plots of the bare GCE (curve a), CDC/GCE (curve b), CuO/GCE (curve c), and CDC@CuO-NPs/GCE (curve d) are presented. The bare GCE (curve a), CuO/GCE (curve c), and CDC@CuO-NPs/GCE exhibit charge-transfer resistance (Rct) values of approximately 117 Ω, 263 Ω, and 167 Ω, respectively. In contrast, the GCE modified with CDC (curve b) shows a significant decrease in Rct along with an almost linear Nyquist profile in the low-frequency region. This behavior indicates the high electrical conductivity of CDC and its ability to facilitate rapid charge transfer. Most importantly, when the CDC@CuO-NP composite is introduced onto the GCE surface (curve d), the Rct value decreases considerably compared to that of CuO-NPs/GCE. This improvement can be attributed to the conductive CDC framework, which enhances electron transfer efficiency by providing intimate interfacial contact between CuO nanoparticles and the carbon matrix. These findings confirm the successful fabrication and superior electrochemical performance of the CDC@CuO-NPs/GCE. The electrochemical active surface area (ECSA) was calculated using the Randles–Sevcik equation: Ip = 2.69 × 10^5^ A D^1/2^ n^3/2^ υ^1/2^ C where A is the electrochemical surface area of the electrode, n is the number of electrons involved in the redox reaction, D is the diffusion coefficient of the molecule in solution (6.67 × 10^−6^ cm^2^ s^−1^ for ferricyanide), C is the concentration of ferricyanide, and υ is the scan rate. The calculated electrochemical surface areas of GCE, CuO/GCE, CDC/GCE, and CDC@CuO-NPs/GCE were 0.0707, 0.1511, 0.1698, and 0.2295 cm^2^, respectively.

### 2.3. The Effect of the Scan Rate

**Figure 5:**
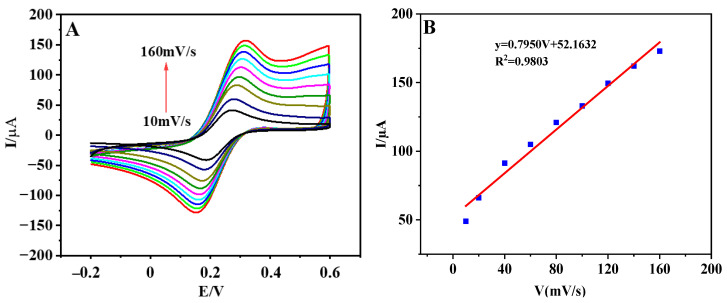
(**A**) Cyclic voltammograms of the CDC@CuO/GCE electrode recorded in 5 mmol L^−1^ K_3_[Fe(CN)_6_]/K_4_[Fe(CN)_6_] containing 0.1 mol L^−1^ KCl at scan rates ranging from 10 to 160 mV s^−1^. (**B**) Linear relationship between the anodic peak current and scan rate.

Figure 5A shows the CV of the CDC@CuO/GCE recorded at different scan rates from 10 to 160 mV s^−1^. As the scan rate increased, both anodic and cathodic peak currents increased gradually, indicating an enhancement of the electrochemical response. The well-defined oxidation and reduction peaks demonstrate the reversible redox behavior of the [Fe(CN)_6_]^3−^/[Fe(CN)_6_]^4^ redox couple at the electrode surface. In addition, in Figure 5B, only a slight shift in the peak potentials was observed as the scan rate increased, suggesting fast electron-transfer kinetics, good electrochemical reversibility, and an adsorption-controlled process at the surface. The proportional increase in peak current with scan rate confirms the effective charge-transfer capability and high electrochemical activity of the CDC@CuO-modified electrode. These results indicate that the CDC@CuO nanocomposite provides a conductive interface that facilitates rapid electron transport and enhances the sensor’s electrochemical performance.

### 2.4. The Electrochemical Activity of DDVP Was Determined by Cyclic Voltammetry and Differential Pulse Voltammetry on the CDC@CuO-NP/GCE Sensor

**Figure 6:**
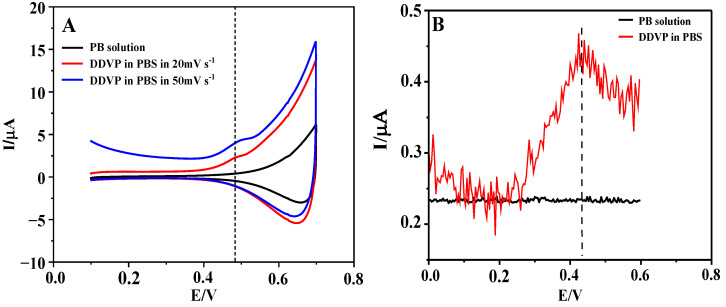
CV (**A**) and DPV (**B**) curves of the CDC@CuO-NPs/GCE recorded in 0.1 M PBS solution pH 6.5 in presence of 100 μM dichlorvos.

The study of electrochemical behavior was conducted in a 0.1 M PBS buffer. As shown in Figure 6A, this buffer served as a background electrolyte in which no electrochemical activity was observed; in other words, no currents resulting from electrochemical reactions were recorded. According to previous studies, the DDVP anode peak is expected at 0.43 V, and this oxidation peak was clearly observed at a concentration of 100 μmol. This indicates that the DDVP pesticide molecule possesses electrochemical properties and that the oxidation occurs at 0.43 V. Furthermore, as the scan rate was increased from 20 mV/s to 50 mV/s, the oxidation peak current increased, thereby confirming that this anodic peak corresponds to the DDVP pesticide. In particular, the DPV results shown in Figure 6B revealed a very low peak current during anodic oxidation. Such a limited and unstable current response indicates that the voltammetric determination of DDVP using CV and DPV lacks sufficient sensitivity for reliable analysis. The corresponding DPV data are provided in the Supplementary Materials [31,32,33,34]. Therefore, due to the low sensitivity and instability of the voltammetric signals, these methods were not used for further analysis. Instead, electrochemical impedance spectroscopy (EIS) was employed as an alternative technique.

### 2.5. Results Analysis of Electrochemical Impedance Spectroscopy

**Figure 7:**
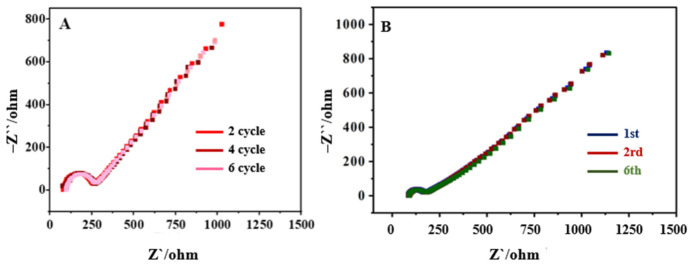
Cyclic voltammograms of bare GCE (**A**) and CDC@CuO/GCE (**B**) electrodes recorded in 5 mmol L^−1^ K_3_[Fe(CN)_6_]/K_4_[Fe(CN)_6_] and 0.1 mol L^−1^ KCl solution (pH 6.5), over six consecutive cycles for stability assessment.

EIS is one of the most important methods for evaluating the electrochemical properties of electrode surfaces. Figure 7A shows the Nyquist plots for a bare glass electrode and an unmodified GCE. As shown, the Nyquist plots exhibit a distinct semicircular resistance shape. The diameter of this semicircle represents the Rct. The characteristics of the curves indicate that the charge-transfer resistance of the pure GCE is relatively high. This result explains the slow reaction rate at the electrode surface and the limited electron transfer. As shown in Figure 7B, the semicircular segment of the resulting curve is only half that of a GCE, indicating a significant decrease in the Rct at the electrode surface and an improvement in the electron transport velocity. Such a low Rct value suggests enhanced conductivity at the electrode surface and higher electrochemical reaction efficiency. The CDC@CuO nanocomposites used in the modification increase the electrode’s surface area and promote electron transport. The CDC is neatly stacked in some areas and amorphously dispersed in others; this structure exhibits high conductivity and improves current transport, whilst the copper oxide nanoparticles act as an electrocatalytic active medium. The synergistic effect of these two nanomaterials enhances the electrochemical performance of the electrode.

**Figure 8:**
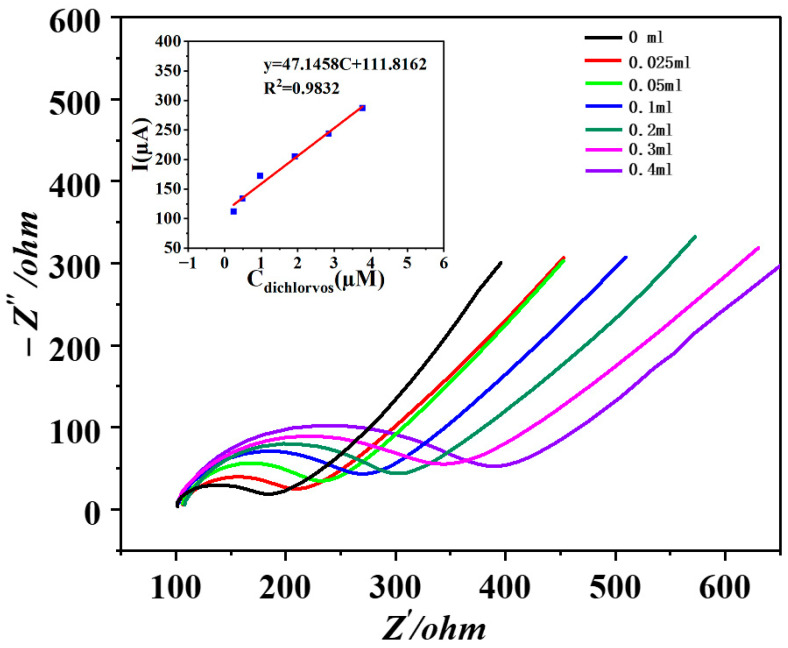
EIS curves of CuO@CDC-NPs/GCE in relation to DDVP pesticide. A total of 0.025 mL–0.4 mL of 100 µmol DDVP solution was added to 5 mM K_3_[Fe(CN)_6_]/K_4_[Fe(CN)_6_] + 0.1 M KCl solution.

Electrochemical impedance spectroscopy (EIS) was employed to investigate the interaction of DDVP with the CDC@CuO-NP/GCE surface. Nyquist plots were recorded in a 5 mM K_3_[Fe(CN)_6_]/K_4_[Fe(CN)_6_] solution containing 0.1 M KCl after the addition of different DDVP concentrations (0.025–0.4 mL). As shown in Figure 8, the diameter of the semicircle in the high-frequency region, corresponding to the charge-transfer resistance (Rct), increased progressively with increasing DDVP concentration. This behavior indicates that DDVP molecules adsorb onto the electrode surface, thereby hindering electron transfer between the redox probe and the electrode interface. Consequently, the charge-transfer resistance increased with increasing DDVP concentration, confirming the successful interaction of DDVP with the CDC@CuO-NP surface. The inset calibration plot demonstrates a linear relationship between the analytical signal and DDVP concentration, described by the equation y = 47.1458C + 111.8162, with a correlation coefficient of R^2^ = 0.9832. The developed sensor exhibited a limit of detection (LOD) of 2.3 nM, demonstrating its high sensitivity toward DDVP. The high linearity and low detection limit confirm the suitability of the CDC@CuO-NPs/GCE for the sensitive electrochemical determination of DDVP. Moreover, compared with the most recently reported electrochemical sensors [33,34,35] for the determination of DDVP, our proposed nanocomposite sensor can achieve ultrasensitive detection of DDVP with a much wider linear range and significantly lower detection limits (Table 1).

## 3. Materials and Methods

### 3.1. Chemicals and Reagents

Petroleum coke, nitrogen gas (N_2_), potassium ferrocyanide (K_4_[Fe(CN)_6_]), potassium ferricyanide (K_3_[Fe(CN)_6_]) copper(II) sulfate pentahydrate (CuSO_4_·5H_2_O), ethanol (EtOH, ≥99.7%), cetyltrimethylammonium bromide (CTAB) and N,N-dimethylformamide (DMF) (98%) were purchased from Sinopharm Chemical Reagent Co., Ltd. (Shanghai, China).

The electrochemical experiments were conducted in a solution of 5 mmol/L K_3_[Fe(CN)_6_]/K_4_[Fe(CN)_6_] and 0.1 M KCl. This redox pair solution is well suited for studying the electrochemical redox behavior of the synthesized material. The pH of the solution was adjusted to the desired value using hydrochloric acid and sodium hydroxide solutions.

### 3.2. Apparatus

Electrochemical measurements, such as cyclic voltammetry (CV) and electrochemical impedance spectra (EIS), were performed using a CS Patentiostat/Galvanostat (Wuhan CorrTest Instruments Co., Ltd., Wuhan, China). A standard three-electrode cell was used, with a glassy carbon electrode (GCE) (surface area 3 mm) as the working electrode, an Ag/AgCl electrode (saturated with KCl) as the reference electrode, and a platinum wire as the counter electrode. All potentials mentioned in the experimental results were referenced to the standard Ag/AgCl (KCl-saturated) reference electrode. The surface morphology of the fabricated films was studied using SEM images obtained on a ZEISS Ultra 55 scanning electron microscope (ZEISS, Carl Zeiss AG, Oberkochen, Germany). A pH meter (BA2204C, Biobase Biometch Co., Ltd., Jinan, Shandong, China) was used to adjust the pH of the buffer solution. TEM and EDS analyses were performed using an Energy-Dispersive Spectrometry system attached to a transmission electron microscope (EDS–TEM, Bruker Nano GmbH, Berlin, Germany). A rotary vacuum tube furnace (BR-16SRT-80/450 Biometch Co., Ltd., Jinan, China) was used for synthesis and thermal treatment of nanomaterials.

### 3.3. Preparation of the CDC@CuO-NP Nanocomposite

**Figure 9:**
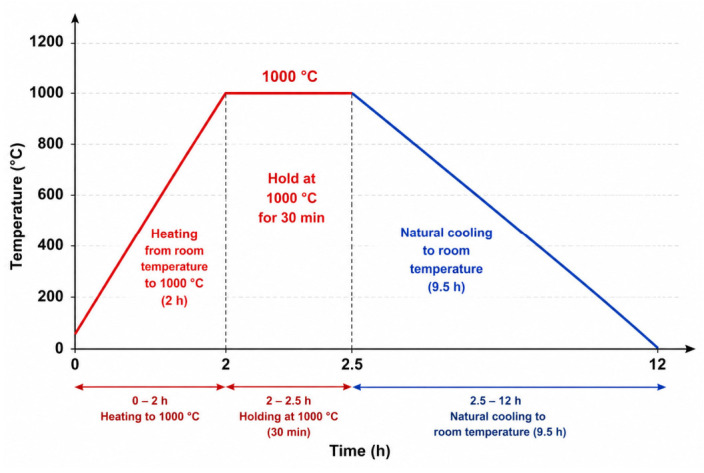
Temperature–time diagram of the heat treatment of petroleum coke.

To prepare CDC, 3 g of petroleum coke was weighed and placed in a rotary vacuum tube furnace. The temperature was gradually raised to 1000 °C and maintained at that level for 12 h. Figure 9 shows the temperature–time diagram of the thermal treatment process for petroleum coke. This heating range is used for the thermal activation of the material or for the formation of the crystalline structure of carbon materials and the development of a porous structure. The temperature is then gradually lowered. The gas flow rate, as indicated by the rotameter, to maintain an inert atmosphere in the reaction chamber during heat treatment, was approximately 30–35 mL/min. Continuous gas supply prevents oxygen from entering the system and oxidizing the material. During the high-temperature process, stable carbon structures formed, and volatile components were completely removed.

**Figure 10:**
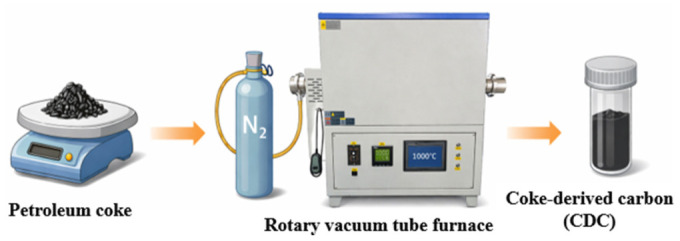
Schematic illustration of CDC synthesis.

After natural cooling, a homogeneous black powder corresponding to CDC was obtained. The synthesized material was subsequently used for further surface modification and the fabrication of nanocomposites (Figure 10).

**Figure 11:**
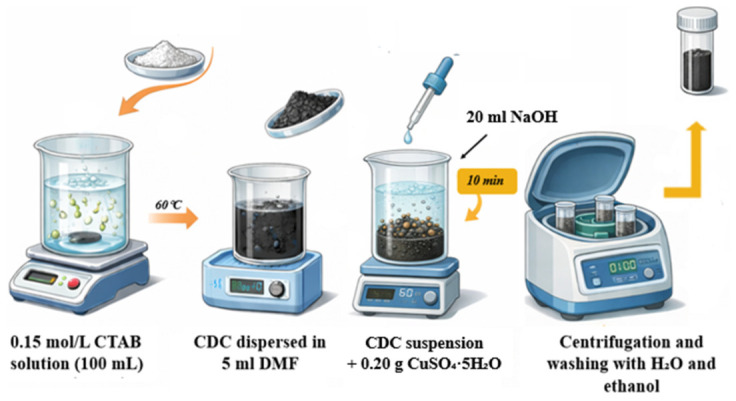
Schematic illustration of the synthesis of CDC@CuO nanocomposite.

To synthesize the CDC@CuO-NP nanocomposite (Figure 11), a 0.15 M solution of cetyltrimethylammonium bromide (CTAB, 100 mL) was first prepared. The CTAB was dissolved in distilled water under magnetic stirring at 60 °C. In a separate flask, 30 mg of CDC was dispersed in 5 mL of dimethylformamide (DMF) and ultrasonicated to obtain a homogeneous black suspension. The resulting CDC suspension was then mixed with 0.20 g of copper(II) sulfate pentahydrate (CuSO_4_·5H_2_O) and CTAB, and the mixture was stirred continuously for 20 min [38]. Then, under continuous stirring, 20 mL of a 0.2 M sodium hydroxide (NaOH) solution was added dropwise to the mixture, resulting in the precipitation of an insoluble powder, presumably coated with copper oxide nanoparticles. The suspension was then stirred for another 10 min. It was left to settle for 30 min to allow the precipitation to complete.

### 3.4. Preparation of Modified Electrodes

The glass carbon electrode (GCE) was sequentially polished with aluminum oxide powder with particle sizes of 1.0, 0.3, and 0.05 microns. The electrode was then subjected to ultrasonic treatment for 3 min using anhydrous ethanol and distilled water. After cleaning, the electrode was dried with nitrogen. Preparation of CDC@CuO/GCE: 9 mg of CDC@CuO was dissolved in a mixture of 1 mL of ethanol and 2 mL of distilled water. The mixture was placed in ultrasound treatment for 30 min to ensure uniform particle distribution. Then, 5 µL of the solution was pipetted onto the surface of a clean glass carbon electrode. The electrode was left to dry at room temperature for 15 min, after which the CDC@CuO/GCE was ready for use in further studies.

### 3.5. The Electrochemical Detection Procedure

Electrochemical studies were carried out using CV, DPV, and EIS methods. The main operating parameters used during the measurements are shown in Table 2.

## 4. Conclusions

In this study, CDC was successfully synthesized from petroleum coke through high-temperature carbonization under a nitrogen atmosphere. Subsequently, a CDC@CuO-NP nanocomposite was fabricated by depositing CuO nanoparticles onto the CDC matrix. SEM, TEM, EDS, and elemental mapping analyses confirmed the successful formation of the composite and the uniform distribution of CuO nanostructures on the carbon surface. Electrochemical characterization revealed that incorporating CuO significantly enhanced CDC’s electrochemical performance by increasing the electroactive surface area and facilitating electron transfer. The synergistic interaction between the conductive CDC framework and catalytically active CuO nanoparticles resulted in improved charge-transfer properties compared with the bare and CDC-modified electrodes. The CDC@CuO-NPs/GCE was further applied for the electrochemical detection of DDVP using EIS. The sensor exhibited a concentration-dependent increase in charge-transfer resistance, indicating effective adsorption of DDVP molecules on the electrode surface. A good linear relationship was obtained from 247 nM to 3770 nM, with the regression equation y = 47.1458C + 111.8162 and a correlation coefficient R^2^ = 0.9832. The developed sensor achieved a low limit of detection (LOD) of 2.3 nM, demonstrating its high sensitivity toward DDVP.

The results indicate that the CDC@CuO-NP nanocomposite is a promising, low-cost, and efficient electrode material for the sensitive detection of organophosphorus pesticides. The proposed sensing platform offers significant potential for environmental monitoring, food safety control, and the development of advanced electrochemical sensors.

## Figures and Tables

**Table 1:** Comparison of performances of the CDC@CuO-NPs/GCE with other modified electrodes.

Electrode	Detection Limit (nM)	Linear Range (nM)	Reference
Acetylcholinesterase/rGO@Nafion/GCE	9.05	22.6–453	[35]
Acetylcholinesterase/chitosan@TiO_2_/rGO/GCE	29	36–22,600	[36]
ChOx/PBCBethaline-HNO_3_ PTD	1.6	2.5–60	[37]
CDC@CuO-NPs/GCE	2.3	247–3770	This work

**Table 2:** Experimental parameters used for electrochemical measurements.

Detection Method	Potential Range	Frequency Range	Scanning Speed	Sampling Interval	Frequency
Cyclic voltammetry	−0.2–+0.6 V	-	50 mV/s	0.01 V	50 Hz
Differential pulse voltammetry	0.0–+0.8 V	-	-	0.1 V	20 Hz
Electrochemical interference spectroscopy (EIS)	-	0.01 Hz–100,000 Hz	-	-	-

## Data Availability

The data presented in this study are available upon request from the corresponding author.

## Supplementary Materials

The following supporting information can be downloaded at: https://www.mdpi.com/article/10.3390/molecules31122129/s1, Figure S1: DPV curves of the CDC@CuO-NPs/GCE recorded in 0.1 M PBS solution pH 8.5, 7.5, 6.5, 5.5 and 4.5 in presence of 400 μM dichlorvos. References [31,32,33,34] are cited in Supplementary Materials.
