# Supplementary material for: Synthesis of Carbon Nanomaterial from Coke and Preparation of Copper Oxide-Based Composite
Source: Molecules. 2026 Jun 17;31(12):2129. doi: 10.3390/molecules31122129 (PMC13305543; doi:10.3390/molecules31122129)
Supplement: Supplementary file 1 [file molecules-31-02129-s001.zip › molecules-4331995-supplementary.pdf]

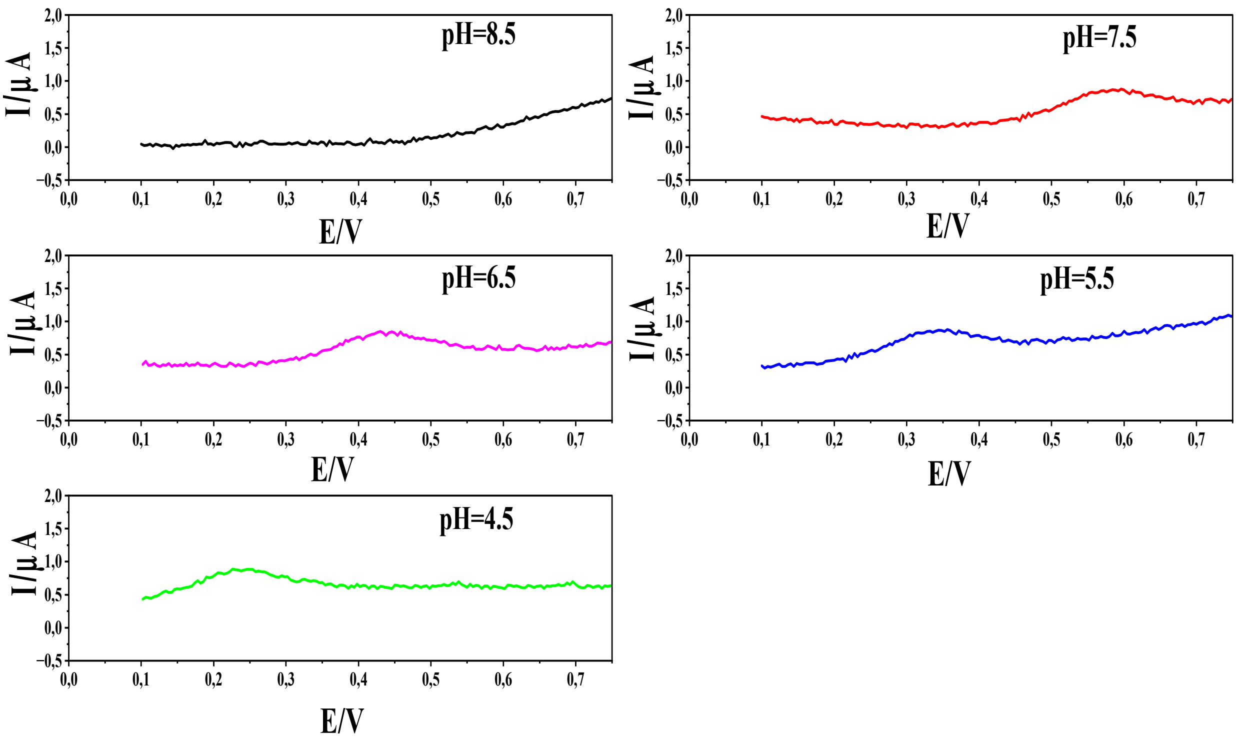

Figure S1. DPV curves of the CDC@CuO-NPs/GCE electrode recorded in 0.1M PBS solution pH 8.5,7.5,6.5,5.5 and 4.5 in presence of 400 µM dichlorvos.

The electrochemical behavior of DDVP at different pH values was investigated in 0.1 M phosphate-buffered saline (PBS). As shown in Figure 1, only a very weak anodic oxidation peak was observed over the studied pH range. Such a low and unstable current response indicates that the direct voltammetric determination of DDVP by differential pulse voltammetry (DPV) does not provide sufficient sensitivity and reproducibility for reliable quantitative analysis. Furthermore, previous studies have reported [1-4] that DDVP (O,O-dimethyl O-(2,2-dichlorovinyl) phosphate) exhibits inherently low electrochemical activity on conventional electrode surfaces, resulting in poor electron-transfer kinetics and weak voltammetric signals. Therefore, direct electrochemical detection of DDVP remains challenging, and alternative approaches such as electrochemical impedance spectroscopy (EIS) may offer improved analytical performance.

References

[1] Vargas, R., Núñez, O., Salazar, R., et al. Electrochemical oxidation of dichlorvos on SnO2 -Sb2O5 electrodes. Applied Catalysis B: Environmental, 2014, 144, 107–111. https://doi.org/10.1016/j.apcatb.2013.06.016

[2] Randviir, E.P.; Banks, C.E. Electrochemical impedance spectroscopy: an overview of bioanalytical applications. Analytical Methods, 2013, 5, 1098–1115. https://doi.org/10.1039/C3AY26476A

[3] Zhang, Y., Liu, X., Wang, J., et al. Emerging Technologies for Degradation of Dichlorvos: A Review. International Journal of Environmental Research and Public Health, 2021, 18, 5789. https://doi.org/10.3390/ijerph18115789

[4] Yu, S.; Wang, Z. Density Functional Theory Insight in Photocatalytic Degradation of Dichlorvos. Toxics, 2024, 12, 928. https://doi.org/10.3390/toxics12120928
